# Chloroform in Obstetrics

**Published:** 1860-03

**Authors:** William Pettigreu

**Affiliations:** Western Medical and Surgical Society


*Chloroform in Obstetrics.* By William Pettigreu, M. D. *Western Medical and Surgical Society.*

As a general rule he deprecated its use in ordinary or natural labor for the following reasons:

1.—That the accoucheur should attend solely and strictly, to his own avocation, and that it therefore necessitates the presence of a second practitioner.

2.—The folly of occurring any risk of asphyxia or death, although such cases in the lying-in room are rare with chloroform in comparison with those in which surgical operations are performed.

3.—That under ordinary circumstances where matters are favorable and progress natural, it tends to depress the system, leaving the entre expulsion of the fœtus to the efforts of the uterus, supplied as it is by organic nerves, while the muscles of animal life which so forcibly assist its action are almost paralyzed.

4.—By its administration there is danger to both mother and child.

Cases illustrative to these objections were related; the exception to the general rule being, where the mother was of a delicate and nervous temperament, and where chloroform was administered in a very modified form, more to attract the attention of the patient from her fears than to lessen the natural throes of labor. In protracted labor the author had experienced much benefit in its administration, and although the pains for the first ten minutes appeared arrested, they afterwards returned more strongly, with greater regularity, and under its use the rigidity relaxed, the mucus became more freely secreted, the countenance of the patient became less anxious, and the pulse quickened at first, beame stronger, and the child was born in a short time. Cases illustrative of the facts were then related.

The author, in his limited experience, bore out the observation of Dr. Simpson, that hæmorrhage seldom or never occurred after the use of chloroform.—*Medical Times and Gazette.*

---

Death of a Medical Practitioner from Chloroform.—Dr. Renwick, a medical practitioner of Alloa, Scotland, died recently under the influence of chloroform, which was administered for the purpose of undergoing an operation on an inverted toe nail. He had, some time previously, suffered from pain in the region of the heart, and it is said that his father died suddenly of the heart disease.

---

Nœvus cured by Creasote.—Dr. Buzalsky reports in the *Medical Zeitung*, the entire removal of a nœvus on a child's temple by penciling twice a day with creasote.

---

The Hospital for Nervous Diseases.—Dr. J. S. Ramskill has been appointed, in connection with Dr. Brown-Sequard, a Physician to the National Hospital for the paralyzed and epileptic.

---

A Suicide by Chloroform occurred lately in Liverpool. Eight ounces of the article were poured into a dish, and the individual died with his head over the vessel.

